# Causal links between circulatory inflammatory cytokines and risk of digestive polyps: a Mendelian randomization analysis

**DOI:** 10.3389/fphar.2024.1405503

**Published:** 2024-10-08

**Authors:** Ziqi Yan, Hongming Zheng, Jieni Feng, Yiting Li, Zhifan Hu, Yuan Wu, Guibin Liao, Taosheng Miao, Zexin Qiu, Qiaolan Mo, Jia Li, Ailin Lai, Yue Lu, Bin Chen

**Affiliations:** ^1^ Department of Gastroenterology, The First Affiliated Hospital of Guangzhou University of Chinese Medicine, Guangzhou, China; ^2^ The First Clinical College, Guangzhou University of Chinese Medicine, Guangzhou, China; ^3^ The Second Clinical College, Guangzhou University of Chinese Medicine, Guangzhou, China; ^4^ The Second Affiliated Hospital of Guangzhou University of Chinese Medicine (Guangdong Provincial Hospital of Chinese Medicine), Guangzhou, China

**Keywords:** gastric polyp, intestinal polyp, gallbladder polyp, GWAS -genome-wide association study, inflammation biomarkers, Mendelian randomization (MR), Bayesian model averaging (BMA)

## Abstract

**Background:**

There is a high morbidity of polyps in the digestive tract, and certain subtypes of polyps are thought to induce cancer progression and often recur, which may be associated with chronic inflammation. Mendelian randomization (MR) can help identify potential causative relationships and inform early treatment action.

**Methods:**

We performed a bidirectional two-sample MR analysis implementing the results from genome-wide association studies for 41 serum cytokines from 8,293 Finnish individuals, and three types of polyps from European ancestry, respectively, including gastric polyp (6,155 cases vs. 341,871 controls), colonic polyp (22,049 cases vs. 332,368 controls) and gallbladder polyp (458 cases vs. 340,083 controls). Inverse-variance weighted (IVW), weight median (WM), and MR-Egger methods were used for calculating causal estimates. Furthermore, Bayesian model averaging MR (MR-BMA) method was employed to detect the dominant causal circulatory cytokines with adjustment for pleiotropy effects.

**Results:**

Our univariable MR using inverse-variance weight method identified causal associations of IL-2ra (OR: 0.892, 95%CI: 0.828–0.961, p = 0.003), MIG (OR: 1.124, 95%CI: 1.046–1.207, p = 0.001) and IL-18 (OR: 0.912, 95%CI: 0.852–0.977, p = 0.008) with gastric polyp, MIP1b (OR: 0.956, 95%CI: 0.927–0.987, p = 0.005) and IL-6 (OR: 0.931, 95%CI: 0.870–0.995, p = 0.035) with colonic polyp and IL-9 (OR: 0.523, 95%CI: 0.345–0.794, p = 0.0007) with gallbladder polyp. Finally, our MR-BMA analysis prioritized MIG (MIP = 0.332, MACE = 0.022; PP: 0.264, MSCE = 0.059), IL-18 (MIP = 0.302, MACE = −0.020; PP: 0.243, MSCE = −0.059) and IL-2ra (MIP: 0.129; MACE: −0.005; PP: 0.112, MSCE: −0.031) for gastric polyp, and MIP1b (MIP = 0.752, MACE = −0.033; PP: 0.665, MSCE = −0.044) and IL-6 (MIP: 0.196; MACE: −0.012; PP: 0.140, MSCE: −0.064) for colonic polyp, and IL-9 (MIP = 0.936, MACE = −0.446; PP: 0.781, MSCE = −0.478) for gallbladder polyp as the top-ranked protective factors.

**Conclusion:**

Our research advances the current understanding of the function of certain inflammatory biomarker pathways in the genesis and malignant mutation of polyps in the digestive tract. Deeper substantiation is necessary to assess the potential of these cytokines as pharmacological or lifestyle targets for digestive polyps prevention.

## 1 Introduction

The liberal use of endoscopy has led to an increased identification of polyps in multiple segments of the digestive tract. Gastric, colorectal, and gallbladder polyps are discovered in as many as 6%, 26.3%, and 5% of patients, respectively (Lin et al., 2008; Wang et al., 2023; Carmack et al., 2009; Wennmacker et al., 2018). Polyps in the digestive are a diverse set of lesions that can be neoplastic or non-neoplastic, epithelial or non-epithelial. Several subtypes, such as intestinal-type and foveolar-type adenoma in the stomach, adenoma in the colon, and gallbladder, are thought to be the precursor lesions of malignant tumors (Atkin et al., 2012; Kővári et al., 2021; Xu et al., 2017). Although the biological features of the hyperplastic polyps are not fully mentioned but studies indicate the hyperplastic polyps as a significant inducer in cancer progression (Jass, 2004; Gibson et al., 2011). Furthermore, polyps often recur, for example, the recurrence colorectal polyp of at a rate of 20%–50% (Hennink et al., 2015). Consequently, it is essential to clarify the underlying causes of polyp formation.

Observational data indicates that chronic inflammatory conditions are linked to a higher likelihood of developing neoplastic polyps, such as gastric and colorectal polyps (Axelrad et al., 2023). It has been postulated that the promotion of inflammation and angiogenesis by 5-lipoxygenase (5-LO)/Leukotriene B4 (LTB4) and the alternation in cell proliferation and metabolism in inflammatory regions by Wnt/Myc signaling have played roles in the formation of gastrointestinal polyps (Leibowitz et al., 2023; Gounaris et al., 2015). Additionally, reagents that inhibit the inflammatory response, including the utilization of phosphodiesterase inhibitor may potentially hinder inflammation in inflammatory bowel disease (IBD) (Khoshakhlagh et al., 2007), thereby playing a protective role in the prevention of colorectal cancer (CRC) and advanced colorectal polyps (Cullinane et al., 2023). On the other hand, activated inflammatory cells have the ability to induce the production of reactive oxygen species and the buildup of reactive nitrogen intermediates in adjacent cells (Grivennikov et al., 2010). These processes can increase mutation rates by directly or indirectly damaging DNA and its protein products, thereby enhancing the formation of mutated cells and potentially leading to the progression of polyps into tumors (Rokavec et al., 2016; Thanan et al., 2012).

Biases like reverse causation, unmeasured confounding, and small sample sizes can affect the findings of observational studies that explore the potential causal link between these circulating inflammatory cytokines and polyps in the stomach, colon, and gallbladder. In Mendelian randomization (MR), hereditary genetic variations serve as instrumental variables to represent exposure, and the inference of causality is less vulnerable to typical confounding factors in comparison to traditional observational studies, such as the environment after birth, socioeconomic position, and behavioral aspects. Furthermore, if a causal link can be proven, using the information from these preclinical connections to assess translatability to digestive tract polyps may have special value in guiding early therapeutic action. To investigate independent and group associations, and identify the most likely causative circulatory cytokines, both univariable MR analysis and multivariable MR analysis using Bayesian model averaging (MR-BMA) are utilized. The identification of potential circulating inflammatory cytokines, which can be detected before clinical symptoms and are involved in the development of digestive polyps, would assist in further investigating early intervention possibilities and personalized treatments.

## 2 Methods

**FIGURE 1:**
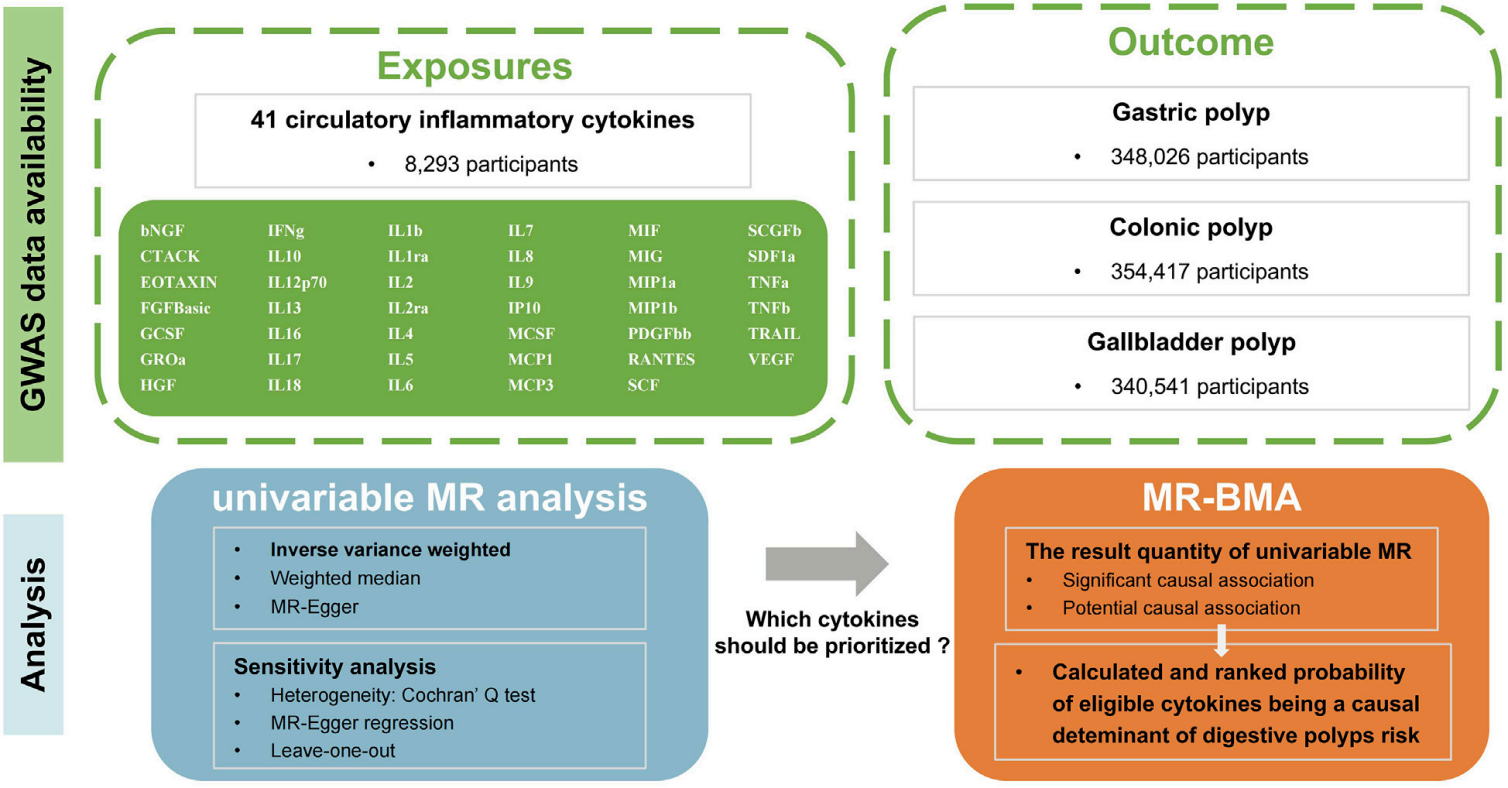
Flow-chart on the overall study design. (MR, Mendelian randomization; MR-BMA, multivariable Mendelian randomization analysis using Bayesian model averaging; GWAS, Genome-wide association Study; bNGF, beta nerve growth factor; CTACK, cutaneous T cell-attracting chemokine; FGFBasic, basic fibroblast growth factor; GCSF, granulocyte colony-stimulating factor; GROa, growthregulated oncogene-a; HGF, hepatocyte growth factor; IFNg, interferon gamma; IL, interleukin; IP, interferon gamma-induced protein 10; MCP1, monocyte chemotactic protein 1; MCP3, monocyte-specific chemokine 3; MCSF, macrophage colony-stimulating factor; MIF, macrophage migration inhibitory factor; MIG, monokine induced by interferon gamma; MIP1a, macrophage inflammatory protein-1a; MIP1b, macrophage inflammatory protein-1b; PDGFbb, platelet-derived growth factor BB; RANTES, regulated upon activation normal T cell expressed and secreted factor; SCF, stem cell factor; SCGFb, stem cell growth factor beta; SDF1a, stromal cell derived factor-1 alpha; SNPs, single-nucleotide polymorphisms; TNFa, tumor necrosis factor alpha; TNFb, tumor necrosis factor beta; TRAIL, TNF-related apoptosis-inducing ligand; VEGF, vascular endothelial growth factor; IBD, inflammatory bowel disease; CRC, colorectal cancer; ICC, intrahepatic cholangiocarcinoma; 5-LO, 5-lipoxygenase; LTB4, Leukotriene B4).

The conceptual framework of our study is summarized in Figure 1. The R platform (version 4.3.1) was utilized for all the analyses. The “TwoSampleMR,” and “ggplot2” packages were utilized for statistical analysis and data visualizations. The MR-BMA was performed using the R-code that was made available on GitHub at the following link: https://github.com/verena-zuber/demo_AMD.

### 2.1 Data source

We used four datasets from publicly available summarized Genome-wide association Study (GWAS) data for this MR analysis. The GWAS summary data for digestive polyps (gastric polyp, colonic polyp, gallbladder polyp) came from the data released by Sakaue et al. (2021), and these were then sorted and stored by the GWAS catalog (https://www.ebi.ac.uk/gwas/). The gastric polyp data came from a meta-analysis research that included 6,155 cases and 341,871 controls of European ancestry of both neoplastic polyps (intestinal-type adenomatous polyps, gastric-type adenomas, and fundic gland polyps) and non-neoplastic polyps (hyperplastic polyps and hamartomatous polyps). For colonic polyp, adenomatous colon polyp, and colon inflammatory polyp are included in the comparison of 22,049 cases and 332,368 controls of European ancestry (GWAS ID: GCST90018827, Trait name: polyp of colon). The summary data for gallbladder polyp obtains mainly 458 cases and 340,083 controls of European ancestry (GWAS ID: GCST90018846, Trait name: polyp of gallbladder). For inflammatory circulatory cytokines, data were gathered from a study testing the association between gene variants and 41 types of cytokines in 8,293 Finnish participants (Ahola-Olli et al., 2017) (data: Cytokines GWAS results - Datasets - data. bris). The exposure group and the outcome group would not overlap in population selection.

We used GWAS summary statistics from already published studies, and each of them has been authorized by the ethics committee at corresponding institutional review board. Thus, informed permission was not necessary, nor was further ethical clearance.

### 2.2 MR

#### 2.2.1 Instrumental variables (IVs) selection

Initially, we used p < 5 × 10^−8^ as the threshold for genome-wide significance in order to find significantly connected SNPs involving circulatory cytokines and digestive polyps. Due to the limited number of SNPs identified for a certain fraction of circulating cytokines as the exposure, a more lenient criteria (p < 5 × 10^−6^) was established. To carry out sensitivity analyses, this approach was employed to increase the available SNPs. Furthermore, in order to prevent linkage disequilibrium, we grouped together these SNPs with a distance of 10,000 kilobases and a correlation coefficient of 0.001 (kb = 10,000, r2 = 0.001). We eliminated the palindromic SNPs because we could not be certain that they were oriented in the same orientation for exposure and outcome. Additionally, the extent of diversity in exposure was assessed by examining the R-squared value of each SNP, and the efficacy of the instrument was tested by applying the F-statistic to limit the effects of weak instrument bias (Pierce et al., 2011; Palmer et al., 2012).

#### 2.2.2 Univariable MR analyses

MR analysis is based on three core assumptions: relevance, independence, and exclusion constraints (Emdin et al., 2017). Simply put, the aforementioned three attributes refer that: i) there is a correlation between the IVs and exposure; ii) there is no linkage between IVs and any confounding factors associated with exposure and outcomes; iii) there is no other way for IVs to affect outcomes except through the effects on exposure. In the stage of univariable MR analyses, we adopt bidirectional investigation approach. We first incorporated a GWAS involving 41 different circulatory cytokines as the variable of exposure, and three separate GWASs focusing on gastric polyp, colonic polyp, and gallbladder polyp as the respective outcomes. The primary analytical approach was the employment of the inverse-variance weighted (IVW) method with different models, based on heterogeneity (Lawlor et al., 2008). MR-Egger (Bowden et al., 2015) and weighted median (Bowden et al., 2016) (WM) approaches were used to re-estimate casual connections with reduced IVW assumptions. The digestive polyps (gastric, colonic, and gallbladder) were set as the exposure, and each circulatory cytokine was set as the outcome, in order to investigate causality in the opposite direction. The above-mentioned analytical workflow was implemented.

Various methods were employed in our study to conduct sensitivity analysis. Initially, the heterogeneity between individual SNP estimations was analyzed using Cochran’s Q test, which provided support for choosing an appropriate analytical method. Upon exceeding 0.05 as a threshold p-value for heterogeneity, the fixed-effects IVW strategy was deemed the primary methodology; otherwise, the random-effects model was used as an alternative. Secondly, we investigated the horizontal pleiotropy of IVs employing the MR-Egger intercept approach. The mean horizontal pleiotropic impact across SNP was evaluated by the intercept in the MR-Egger test, and the IVW estimate may be biased if the p-value was below 0.05 (Bowden et al., 2015). Next, we conducted a sensitivity analysis by leave-one-out analyses to evaluate the impact of a single SNP on the results. Additionally, funnel and forest plots were created in order to directly assess the presence of pleiotropy.

### 2.3 MR-BMA analysis

Given the significant associations between various circulatory cytokines in terms of sharing numerous genetic variations, it is imperative to consider the effects of “measured pleiotropy.” Furthermore, univariable MR analysis appears powerless in comparing the priority of risk factors. Thus, we utilized Bayesian model averaging (BMA) to further confirm the circulatory cytokines exhibiting enrichment of significance to digestive polyps in univariable MR analysis (IVW p value < 0.05) (Ren et al., 2023; Wang et al., 2022). For more information on the MR-BMA method, please refer to the comprehensive details provided elsewhere (Zuber et al., 2020). In essence, MR-BMA was constructed using multiple standard multivariable MRs, incorporating a subset of randomly selected risk variables, and subsequently implementing the following steps:

(i) Ranking multivariable models: Prioritizing different multivariable models has been accomplished through the posterior probabilities (PPs) of BMA. Furthermore, the MV-MR model provides model-specific causal estimates (MSCE) that indicate the impact of a particular risk factor on the outcome for an individual in the multivariable model.
(ii) Identifying prioritized circulatory cytokines: Prioritizing risk factors based on marginal inclusion probability (MIP) (sum of PPs of all models with the risk factor). Meanwhile, a model-averaged causal estimate (MACE) was also computed, indicating the calculated direct (independent) influence of circulatory cytokines x on outcome y, averaged across each PP. It is worth mentioning that MACE will be skewed toward the null owing to shrinkage done in variable selection (Zuber et al., 2020).
(iii) Sensitivity analyses: This step includes the ability to identify instrument outliers using the Q-statistic (which measures the heterogeneity by comparing observed and predicted associations with outcomes) and significant observations qualified by Cook’s distance (Cd) to pinpoint the instruments that were affecting the accuracy and association with the outcome. SNPs with Q-statistic greater than 10 or Cd greater than 0.19 (4/total SNP N) were marked as flagged, and MR–BMA was repeated without the omitted SNP(s). After eliminating potential outliers, the associations between circulatory cytokines and digestive polyps that remained were deemed to be more dependable.

The z-value for all BMA analyses was 10,000, the prior probability was 0.1, and the prior variance (σ2) was 0.25.

## 3 Results

### 3.1 Univariable MR analyses between circulatory cytokines and digestive polyps

Out of the 41 categories of circulatory cytokines, 17 had at least one genome-wide significant SNP, however when the higher cut-off (p < 5 × 10^−6^) was utilized, all 41 categories had at least one SNP. Our analyses included all of these SNPs. The F-statistics for all variables exceeded 10, suggesting that the findings were less susceptible to the influence of weak instrument bias.

**FIGURE 2:**
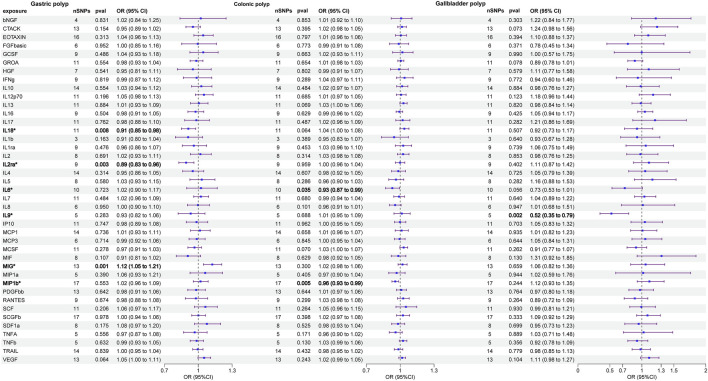
Causal correlations of 41 inflammatory cytokines on gastric polyp, colonic polyp, and gallbladder polyp (the results from inverse variance weighted method were shown for all cytokines). The change in the odds ratio (OR) of risk of gastrointestinal system polyps per one-SD rise in the cytokine level is shown by OR and 95%confidence interval.

The primary results of the univariable MR analyses are presented in Figure 2 and Table 1. The IVW method revealed a potential link between genetically determined higher interleukin-2 receptor subunit alpha (IL-2ra) levels (one-SD increase) and a suggestive 11% reduction in the likelihood of developing gastric polyps (OR: 0.892, 95%CI: 0.828–0.961, p = 0.003). The discovery exhibited resemblance to the WM method (OR: 0.846, 95%CI: 0.768–0.932, p = 0.001) and MR Egger (OR: 0.856, 95%CI: 0.763–0.962, p = 0.003). Through the IVW method, we provided evidence of a causal association between monokine induced by interferon gamma (MIG) and gastric polyp (OR: 1.124, 95%CI: 1.046–1.207, p = 0.001). Although the WM method (OR: 1.099, 95%CI: 0.993–1.217, p = 0.068) and MR Egger (OR: 1.086, 95%CI: 0.935–1.263, p = 0.304) did not identify a statistically significant correlation, they both suggested a comparable pattern of change. A similar cause-and-effect connection was noted in interleukin-18 (IL-18) involving gastric polyp (IVW: OR: 0.912, 95%CI: 0.852–0.977, p = 0.008) (Table 1). Setting gastric polyp as exposure, we demonstrated that gastric polyp is positively causally associated with beta nerve growth factor (bNGF, Beta: 0.016, 95%CI: 0.001–0.030, p = 0.031), basic fibroblast growth factor (FGFBasic, Beta: 0.010, 95%CI: 0.000–0.020, p = 0.048), granulocyte colony-stimulating factor (GCSF, Beta: 0.013, 95%CI: 0.002–0.023, p = 0.019), growthregulated oncogene-a (GROa, Beta: 0.019, 95%CI: 0.004–0.033, p = 0.010), interleukin-13 (IL-13, Beta: 0.018, 95%CI: 0.004–0.033, p = 0.011), interleukin-5 (IL-5, Beta: 0.018, 95%CI: 0.003–0.032, p = 0.017), interleukin-7 (IL-7, Beta: 0.016, 95%CI: 0.001–0.030, p = 0.0432), macrophage colony-stimulating factor (MCSF, Beta: 0.024, 95%CI: 0.006–0.041, p = 0.008) and stem cell growth factor beta (SCGFb, Beta: 0.0014, 95%CI: 0.000–0.028, p = 0.050) levels through IVW (Table 1; Supplementary Table S1).

**TABLE 1:** Results of the univariable MR analyses.

Analysis	OR	Lower 95%CI	Upper 95%CI	P	SNPs, n	Horizontal pleiotropy		Heterogeneity	
(exposure vs. outcome)						Egger intercept	P	Q	P
MIG vs. gastric polyp									
MR Egger	1.086	0.935	1.263	0.304	13	0.010	0.623	12.011	0.363
Weighted median	1.099	0.993	1.217	0.068					
Inverse variance weighted	1.124	1.046	1.207	0.001					
IL-2ra vs. gastric polyp									
MR Egger	0.856	0.763	0.962	0.034	9	0.012	0.394	6.465	0.487
Weighted median	0.846	0.768	0.932	0.001					
Inverse variance weighted	0.892	0.828	0.961	0.003					
IL-18 vs. gastric polyp									
MR Egger	0.962	0.849	1.089	0.555	11	−0.015	0.340	3.033	0.963
Weighted median	0.933	0.848	1.027	0.157					
Inverse variance weighted	0.912	0.852	0.977	0.008					
IL-6 vs. colonic polyp									
MR Egger	0.932	0.816	1.064	0.328	10	0.000	0.981	2.362	0.968
Weighted median	0.932	0.856	1.015	0.107					
Inverse variance weighted	0.931	0.870	0.995	0.035					
MIP1b vs. colonic polyp									
MR Egger	0.974	0.927	1.023	0.304	17	−0.006	0.378	7.947	0.926
Weighted median	0.954	0.916	0.993	0.021					
Inverse variance weighted	0.956	0.927	0.987	0.005					
IL-9 vs. gallbladder polyp									
MR Egger	0.470	0.152	1.456	0.282	5	0.023	0.855	1.321	0.724
Weighted median	0.552	0.327	0.932	0.026					
Inverse variance weighted	0.523	0.345	0.794	0.002					
gastric polyp vs. bNGF									
MR Egger	1.012	0.997	1.028	0.138	20	0.010	0.194	12.952	0.794
Weighted median	1.014	0.997	1.032	0.096					
Inverse variance weighted	1.016	1.001	1.030	0.031					
gastric polyp vs. FGFBasic									
MR Egger	1.012	1.001	1.022	0.045	21	−0.004	0.406	17.312	0.569
Weighted median	1.010	0.998	1.023	0.102					
Inverse variance weighted	1.010	1.000	1.020	0.048					
gastric polyp vs. GCSF									
MR Egger	1.013	1.001	1.025	0.041	21	−0.001	0.899	23.720	0.207
Weighted median	1.013	1.000	1.025	0.051					
Inverse variance weighted	1.013	1.002	1.023	0.019					
gastric polyp vs. GROa									
MR Egger	1.023	1.007	1.038	0.010	20	−0.010	0.174	11.636	0.865
Weighted median	1.020	1.001	1.040	0.041					
Inverse variance weighted	1.019	1.004	1.033	0.010					
gastric polyp vs. IL-13									
MR Egger	1.021	1.005	1.036	0.016	20	−0.006	0.441	8.150	0.976
Weighted median	1.019	1.000	1.039	0.050					
Inverse variance weighted	1.019	1.004	1.033	0.011					
gastric polyp vs. IL-5									
MR Egger	1.018	1.002	1.034	0.038	20	0.000	0.997	17.658	0.478
Weighted median	1.018	1.000	1.037	0.056					
Inverse variance weighted	1.018	1.003	1.033	0.017					
gastric polyp vs. IL-7									
MR Egger	1.014	0.998	1.029	0.103	20	0.006	0.398	17.162	0.512
Weighted median	1.015	0.997	1.034	0.104					
Inverse variance weighted	1.016	1.001	1.031	0.032					
gastric polyp vs. MCSF									
MR Egger	1.020	1.001	1.039	0.051	20	0.011	0.231	18.112	0.448
Weighted median	1.023	1.000	1.047	0.050					
Inverse variance weighted	1.024	1.006	1.042	0.008					
gastric polyp vs. SCGFb									
MR Egger	1.013	0.998	1.028	0.114	21	0.004	0.595	10.887	0.928
Weighted median	1.014	0.996	1.032	0.127					
Inverse variance weighted	1.014	1.000	1.028	0.050					
colonic polyp vs. CTACK									
MR Egger	0.945	0.715	1.251	0.696	77	0.012	0.261	77.265	0.406
Weighted median	1.102	0.960	1.265	0.167					
Inverse variance weighted	1.101	1.003	1.210	0.044					
colonic polyp vs. MIF									
MR Egger	0.806	0.608	1.068	0.138	77	0.006	0.589	62.796	0.842
Weighted median	0.940	0.820	1.077	0.371					
Inverse variance weighted	0.868	0.790	0.953	0.003					

OR, odds ratio; CI, confidence interval; SNPs, single-nucleotide polymorphisms.

The two circulatory cytokines causally associated with colonic polyp were: macrophage inflammatory protein-1b (MIP1b, IVW—OR: 0.956, 95%CI: 0.927–0.987, p = 0.005; WM—OR: 0.954, 95%CI: 0.916–0.993, p = 0.021) and interleukin-6 (IL-6, IVW—OR: 0.931, 95%CI: 0.870–0.995, p = 0.035) (Table 1). In addition, the IVW method findings indicated a significant association between colonic polyp and increased levels of cutaneous T cell-attracting chemokine (CTACK, Beta: 0.097, 95%CI: 0.003–0.190, p = 0.044) and macrophage migration inhibitory factor (MIF, Beta: −0.142, 95%CI: −0.236–0.048, p = 0.003), when considering the inflammatory circulatory cytokines as the outcome (Table 1; Supplementary Table S2).

We identified a credible correlation between circulating interleukin-9 (IL-9) levels and gallbladder polyp risk in univariable MR analysis (IVW—OR: 0.523, 95%CI: 0.345–0.794, p = 0.0007; WM—OR: 0.552, 95%CI: 0.327–0.932, p = 0.026; MR Egger—OR: 0.470, 95%CI: 0.152–1.456, p = 0.252). When the gallbladder polyp was viewed as the exposure, no significant causal relationship was found for gallbladder polyp and any inflammatory circulatory cytokines (Table 1; Supplementary Table S3).

**FIGURE 3:**
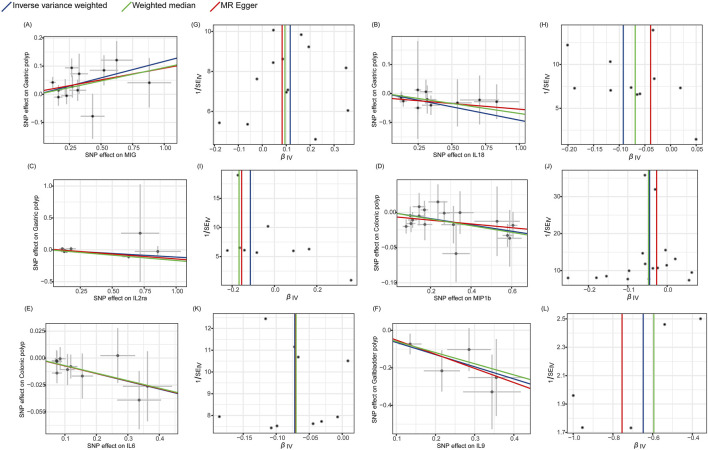
Scatter plots and funnel plots of Mendelian randomization (MR) analyses for significant cytokines in digestive polyp risk. **(A–F)** Individual inverse variance (IV) associations with cytokine risk are displayed versus individual IV associations with gastric polyp, colonic polyp, and gallbladder polyp in black dots, respectively. The 95%CI of odd ratio for each IV is shown by vertical and horizontal lines. The slope of the lines represents the estimated causal effect of the MR methods. **(G–L)** The funnel plots show the inverse variance weighted MR estimate of each cytokine single-nucleotide polymorphism with polyp versus 1/standard error (1/SEIV).

Finally, the Cochran’s Q statistic indicated no heterogeneity in the associations of the above six cytokines (Figure 3; Supplementary Table S4). Thus, in this MR analysis, we employed the fixed-effects IVW approach as the primary analytical technique. Moreover, the MR-Egger regression intercept did not reveal any indication of horizontal pleiotropy (p > 0.05) (Table 1; Supplementary Table S5). Furthermore, the leave-one-out approach demonstrated that a solitary SNP had no effect on the potential causative relationship between significant circulatory cytokines and the risk of digestive polyps (Supplementary Table S6).

### 3.2 MR-BMA between circulatory cytokines and digestive polyps

In the MR-BMA analysis, we incorporated MIG, MIP1b, IL-2ra, IL-6, IL-9, and IL-18, all of which showed significant associations with three digestive polyps in the univariable MR analysis, respectively. We selected sixty-seven instruments that were significantly associated with the above six kinds of circulatory cytokines and the three digestive polyps. Since this is a Bayesian approach, it is not possible to obtain frequentist P values. Alternatively, deductions can be drawn by considering posterior probabilities and evaluating ranking effectiveness.

IL-2ra (MIP = 0.642, MACE = −0.061) and MIG (MIP = 0.237, MACE = 0.019) were the top two circulatory cytokines in sequence prioritized for gastric polyp based on their MIPs, while all other cytokines had MIPs less than 0.2 (Table 2; Supplementary Table S7). The three highest-ranked individual models showed the presence of these identical two cytokines, with “IL-2ra,” “IL-2ra, MIG,” and “MIG” having model-based PP of 0.499, 0.113, and 0.107, respectively (Table 2; Supplementary Table S7). The variant rs12722497 was identified as the most influential based on the best individual models, where the minimum Q-statistic was greater than 10 and Cook’s distance was greater than 0.09 (6/67) (Table 2; Supplementary Figure 1). After removing the outlier rs12722497 (n = 66), the top three prioritized circulatory cytokines were: MIG (MIP = 0.642) with a risk-increasing effect (MACE = 0.022), IL-18 (MIP = 0.302) with a protective effects effect (MACE = −0.020), IL-2ra (MIP = 0.129, MACE = −0.005). MIG, the highest-ranked risk-enhancing factor (PP = 0.264), formed the most optimal individual model in the end (MSCE = 0.059). The second one consisted of IL-18 (PP = 0.243, MSCE = −0.059). In addition to these top two models, three more individual models were given priority based on their PP > 0.01 (Table 2).

**TABLE 2:** Ranking of risk and protective factors for gastric polyp, colonic polyp, and gallbladder polyp. (A) according to their marginal inclusion probability (MIP) and (B) the best 10 individual models according to their posterior probability (PP).

(A) Model averaging			
Disease	Risk/protective factor	MIP	MACE
Gastric polyp			
	MIG	0.332	0.022
	IL-18	0.302	−0.02
	IL-2ra	0.129	−0.005
	IL-6	0.126	0.001
	IL-9	0.122	−0.003
	MIP1b	0.076	0.001
Colonic polyp			
	MIP1b	0.752	−0.033
	IL-6	0.196	−0.012
	IL-18	0.111	0.004
	MIG	0.026	0.001
	IL-9	0.02	0
	IL-2ra	0.014	0
Gallbladder polyp			
	IL-9	0.937	−0.446
	IL-6	0.106	−0.029
	MIG	0.037	0.004
	MIP1b	0.035	0.003
	IL-2ra	0.032	0.002
	IL-18	0.025	0

(B) Individual averaging				
Disease		Risk factors	PP	MSCE
Gastric polyp				
	1	MIG	0.264	0.059
	2	IL-18	0.243	−0.059
	3	IL-6	0.115	0.004
	4	IL-2ra	0.112	−0.031
	5	IL-9	0.111	−0.025
	6	MIP1b	0.07	0.018
	7	IL-18, MIG	0.047	−0.098, 0.095
	8	IL-2ra, MIG	0.01	−0.082, 0.086
	9	IL-9, MIG	0.004	−0.037, 0.062
	10	IL-6, MIG	0.003	−0.02, 0.062
Colonic polyp				
	1	MIP1b	0.665	−0.044
	2	IL-6	0.14	−0.064
	3	IL-18	0.051	0.036
	4	IL-18, MIP1b	0.036	0.037, −0.045
	5	IL-6, MIP1b	0.026	−0.05, −0.039
	6	IL-18, IL-6	0.018	0.045, −0.077
	7	IL-9	0.013	−0.019
	8	MIG	0.012	0.019
	9	MIG, MIP1b	0.009	0.025, −0.046
	10	IL-2ra	0.007	0.009
Gallbladder polyp				
	1	IL-9	0.781	−0.478
	2	IL-6	0.048	−0.375
	3	IL-6, IL-9	0.045	−0.155, −0.408
	4	IL-9, MIG	0.029	−0.505, 0.1
	5	IL-9, MIP1b	0.026	−0.486, 0.088
	6	IL-2ra, IL-9	0.025	0.079, −0.512
	7	IL-18, IL-9	0.018	−0.008, −0.477
	8	IL-18	0.002	−0.075
	9	IL-6, MIP1b	0.002	−0.42, 0.126
	10	MIP1b	0.002	0.069

The three highest-ranked causal circulatory cytokines for colonic polyp were: MIP1b (MIP = 0.752, MACE = −0.033), IL-6 (MIP = 0.196, MACE = −0.012), IL-18 (MIP = 0.112, MACE = 0.004). The first two of them were present within the best two final individual modes: “MIP1b” (PP = 0.665, MACE = −0.044), “IL-6” (PP = 0.14, MACE = −0.064), while other modes’ PPs all being <0.01 (Table 2).

The results indicated that IL-9 was the most highly rated causative circulatory cytokine (MIP = 0.936, MACE = −0.446), with IL-6 coming in second (MIP = 0.107, MACE = −0.029). Only one individual model’s PP > 0.01, consisted of IL-9 (PP = 0.781, MACE = −0.478) (Table 2). No outliers and influential variants were detected for sensitivity analysis in colonic polyp and gallbladder polyp (Supplementary Figures 2, 3).

## 4 Discussion

Research reveals that when compared to healthy controls, individuals with digestive polyps have a unique circulatory cytokine profile (Hopkins et al., 2012), suggesting that circulatory cytokine can be used as potential biomarkers. Therefore, understanding the interactions between early-stage risk factors and prospective biomarkers can enhance our diagnostic accuracy and assist in identifying targets for treatment, particularly if a causal link can be established. Our study discoverd the causative relationships when several inflammatory-related cytokines, including MIG, IL-2ra, IL-8, IL-6, MIP1b, and IL-9, were seen as exposures and three digestive polyps as the outcome through univariable MR analysis and MR-BMA. Through bidirectional investigation approach, it could be inferred that certain biomarkers are more likely to be found later in the course of the illness, like bNGF, FGFBasic, GCSF, GROa, IL-13, IL-5, IL-7, MCSF, SCGFb in gastric polyps, and CTACK, MIF in colonic polyps. No bidirectional causal relationship was found between a single biomarker and polyps in the digestive tract.

In both univariable analyses and MR-BMA, we found inverse associations of IL-2ra and IL-18 and a positive association of MIG with gastric polyp. MIG played the most prioritized effect role, following by IL-2ra and IL-18. MIG was found to possess the greatest causal impact among them. In a research, it was found that reducing the levels of MIG in serum helped improve the damage to the stomach lining caused by *Helicobacter pylori* infection (Eck et al., 2000; Yang et al., 2020), a condition often linked to gastric polyps (Sonnenberg and Genta, 2015; Carmack et al., 2009). Endothelial cells of gastric mucosal vessels and mononuclear cells at locations with T cell infiltration expressed MIG, which attracted inflammatory T cells and develop mucosal injury in *H. pylori* infection (Morey et al., 2018; Eck et al., 2000). Protective effects of IL-18 on the gastrointestinal were previously revealed by multiple studies (Lei-Leston et al., 2017; Nowarski et al., 2015). A study demonstrated that the nucleotide-binding oligomerisation domain 1 (NOD1) molecule facilitates the processing and maturation of IL-18 in epithelial cells through its interactions with caspase-1, thus aiding in the maintenance of intestinal epithelial homeostasis (Tran et al., 2023). Another animal study indirectly supports such an inverse association by the detection that proton pump inhibitors (PPIs) could antagonize the effect of IL-18 (Bertoni et al., 2020), as PPIs are commonly associated with the formation of fundic gland polyps (FGPs) (Sonnenberg and Genta, 2015). Besides, limited information is available regarding the roles of IL-2ra in the process of gastric polyp. For downstream inflammatory factors, our findings were supported by previous studies that demonstrated a strong association between pre-neoplastic alterations and the development of gastric cancer, involving various inflammatory regulators including bNGF, GCSF, IL-13, IL-5, IL-7, and MCSF(Yu et al., 2023; Hayakawa et al., 2017; Morris et al., 2014; Petersen et al., 2018; Wang et al., 2017). Although there have been reports of associations with predisposing factors, there is currently limited epidemiological and experimental evidence to directly investigate the correlation between circulatory cytokines and gastric polyp.

Regarding for colonic polyp, the two prioritized protective factors we identified were IL-6 and MIP1b, respectively. Our findings are consistent with the experimental results of peers, who found that IL-6 plays a role in the impairment of the integrity of the intestinal epithelial barrier in inflammatory situations by enhancing the expression of claudin-2 and increasing the permeability of the intestinal epithelial layer to small substances (Suzuki et al., 2011; Al-Sadi et al., 2014). In contrast to our results, certain studies suggested that higher levels of IL-6 in the serum are linked to the existence of colorectal adenoma (Godos et al., 2017; Sasaki et al., 2012). An animal study observed that the size of colonic polyps decreased significantly when IL-6 expression was absent (Poffenberger et al., 2018). A reason for this discrepancy could be the multiple subtypes of colonic polyp. Another reason could be the potential dual effect that IL-6 might have during various stages. Several studies detect that, under physiological conditions, IL-6 maintains intestinal homeostasis and promotes wound healing after intestinal injury (Kuhn et al., 2018; Kuhn et al., 2014). In reverse MR analysis, it supports that colonic polyp led to a reduced expression of MIF and a rise in CTACK. In a similar manner to our discoveries, CTACK is expressed at high levels in cancer cells that have the ability to metastasize (Karnezis et al., 2019), and its increased concentrations were linked to a statistically significant rise in colorectal cancer risk (Song et al., 2018). MIF has been found to be aberrantly expressed in a number of human solid tumors, including CRC (Li et al., 2023), which contradicts our findings. The reason for the difference may be that we chose colonic polyps as research objects, which reflects the early stage of Inflammation-to-Cancer Transition. *In vivo* experiments have demonstrated that MIF plays a protective role against tumour initiation linked to inflammation (Klemke et al., 2021).

Additionally, we discovered inverse correlations between IL-9 and gallbladder polyp. The limitation to mention is that a relatively small but the largest sample size for gallbladder polyps up to the time of our analysis, might have compromised validity, despite the fact that the aforementioned association was indirectly supported by other studies. The multifunctional cytokine IL-9, which is generated by type 2 helper T cells and mast cells, can act as a positive or negative modulator of immune responses (Tete et al., 2012; Goswami and Kaplan, 2011). Largely experimental studies suggest that IL-9 is positively associated with limiting cancer outgrowth (Almeida et al., 2020; Ma et al., 2018). Additionally, two animal studies offered insights into the relationship between IL-9 and gallbladder polyps. IL-9 was found to stimulate the growth of Interstitial cells of Cajal (ICC) and support its functions (Gong et al., 2014; Ye et al., 2006), potentially resulting in enhanced gallbladder motility and decreased cholestasis, which helps to reduce the occurrence of gallbladder polyps (Vila et al., 2018). However, observational evidence on IL-9 and gallbladder polyp even biliary intraepithelial neoplasia was limited to date. Future research with larger sample sizes is warranted to further validate these findings and explore the underlying mechanisms in greater detail.

The advantage of our study lies in the inclusion of a wide array of inflammatory cytokines in our investigations, as well as the utilization of GWAS from a substantial sample size for the primary analyses. By utilizing knowledge from preclinical associations between circulatory cytokines and gastric polyp, colonic polyp, and gallbladder polyp, this groundbreaking approach provides valuable insights into causally relevant cytokines that could be beneficial in preclinical settings. In addition, our method of instrument selection minimizes the possibility of horizontal pleiotropy by utilizing variations that are close proximal to the encoding gene area (Davey and Hemani, 2014). Additionally, by employing bidirectional MR, relationships between diseases and risk factors were examined in both directions, allowing for further estimation of the upstream and downstream cytokines. The utilization of MR-BMA additionally facilitated the consideration of associations among circulatory cytokines and the formulation of multivariable models of combined cytokines. Moreover, the incorporation of sensitivity analyses in both univariable and multivariable models enabled a more thorough examination of MR assumptions, guaranteeing the investigation of any significant alterations in outcomes.

Recognizing the limitations of our study is equally important. It is important to note that MR impact estimates reflect long-term serum circulatory cytokine concentration and may not fully indicate the extent of the benefit from shorter-term change. However, MR provides a valuable opportunity to validate effects. Besides, the anticipated cause-and-effect relationship can provide insights into potential effectiveness, which can be formally examined in subsequent animal studies and clinical trials. Additionally, the research was restricted to individuals of European descent because of the accessibility of genetic information, potentially constraining the applicability of the findings. Furthermore, prior research has identified variations in immunohistochemistry among different subtypes of polyps in the digestive tract. For example, gastric polyp contains hyperplastic polyps, intestinal-type adenoma, foveolar-type adenoma, and fundic gland polyps, which have different expression profiles of intestinal biomarkers (Kővári et al., 2021). The GWAS conducted on three polyps in the digestive system in our study relied on descriptive diagnosis. This implies that the interpretation of our results was constrained due to the absence of comprehensive phenotyping based on etiology and clinical presentation. The data could not distinguish between the possible effects of circulatory cytokines on the aforementioned single polyp subtypes. And it was necessary to conduct extensive, well-planned genetic investigations with clearly defined subgroups of digestive system polyps as the end result.

## 5 Conclusion

We used MR analysis to investigate the relationship between 41 circulatory cytokines and gastric, colonic, and gallbladder polyps. Using univariable MR analyses and Bayesian model averaging, we identified MIG, IL-18, and IL-2ra as the top three causes of gastric polyp, MIP1b and IL-6 as the top two protective factors for colonic polyp, and an inverse association between IL-9 and gallbladder polyp. In addition, we investigated the source and downstream components of the disease using a bidirectional exploration strategy. Additional human and animal experimental researches are required to determine the potential of these cytokines as therapeutic preventive and therapy options for digestive system polyps.

## Data Availability

The original contributions presented in the study are included in the article/Supplementary Material, further inquiries can be directed to the corresponding authors.

## References

[B1] Ahola-Olli A. V. Würtz P. Havulinna A. S. Aalto K. Pitkänen N. Lehtimäki T. (2017). Genome-wide association study identifies 27 loci influencing concentrations of circulating cytokines and growth factors. Am. J. Hum. Genet. 100 (1), 40–50. 10.1016/j.ajhg.2016.11.007 27989323 PMC5223028

[B2] Almeida R. R. Vieira R. S. Castoldi A. Terra F. F. Melo A. Canesso M. (2020). Host dysbiosis negatively impacts IL-9-producing T-cell differentiation and antitumour immunity. Br. J. Cancer. 123 (4), 534–541. 10.1038/s41416-020-0915-6 32499569 PMC7434765

[B3] Al-Sadi R. Ye D. Boivin M. Guo S. Hashimi M. Ereifej L. (2014). Interleukin-6 modulation of intestinal epithelial tight junction permeability is mediated by JNK pathway activation of claudin-2 gene. PLoS One 9 (3), e85345. 10.1371/journal.pone.0085345 24662742 PMC3963839

[B4] Atkin W. S. Valori R. Kuipers E. J. Hoff G. Senore C. Segnan N. (2012). European guidelines for quality assurance in colorectal cancer screening and diagnosis. First Edition--Colonoscopic surveillance following adenoma removal. Endoscopy 44 (Suppl. 3), SE151–63. 10.1055/s-0032-1309821 23012119

[B5] Axelrad J. E. Olén O. Söderling J. Roelstraete B. Khalili H. Song M. (2023). Inflammatory bowel disease and risk of colorectal polyps: a nationwide population-based cohort study from Sweden. J. Crohns Colitis. 17 (9), 1395–1409. 10.1093/ecco-jcc/jjad056 36994851 PMC10588773

[B6] Bertoni A. Carta S. Baldovini C. Penco F. Balza E. Borghini S. (2020). A novel knock-in mouse model of cryopyrin-associated periodic syndromes with development of amyloidosis: therapeutic efficacy of proton pump inhibitors. J. Allergy Clin. Immunol. 145 (1), 368–378. 10.1016/j.jaci.2019.05.034 31194989

[B7] Bowden J. Davey S. G. Burgess S. (2015). Mendelian randomization with invalid instruments: effect estimation and bias detection through Egger regression. Int. J. Epidemiol. 44 (2), 512–525. 10.1093/ije/dyv080 26050253 PMC4469799

[B8] Bowden J. Davey S. G. Haycock P. C. Burgess S. (2016). Consistent estimation in mendelian randomization with some invalid instruments using a weighted median estimator. Genet. Epidemiol. 40 (4), 304–314. 10.1002/gepi.21965 27061298 PMC4849733

[B9] Carmack S. W. Genta R. M. Schuler C. M. Saboorian M. H. (2009). The current spectrum of gastric polyps: a 1-year national study of over 120,000 patients. Am. J. Gastroenterol. 104 (6), 1524–1532. 10.1038/ajg.2009.139 19491866

[B10] Cullinane C. Brett A. Devane L. Mccullough P. W. Cooke F. Neary P. (2023). The protective role of phosphodiesterase inhibitors in preventing colorectal cancer and advanced colorectal polyps: a systematic review and meta-analysis. Colorectal Dis. 25 (10), 1949–1959. 10.1111/codi.16724 37635321

[B11] Davey S. G. Hemani G. (2014). Mendelian randomization: genetic anchors for causal inference in epidemiological studies. Hum. Mol. Genet. 23 (R1), R89–R98. 10.1093/hmg/ddu328 25064373 PMC4170722

[B12] Eck M. Schmausser B. Scheller K. Toksoy A. Kraus M. Menzel T. (2000). CXC chemokines Gro(alpha)/IL-8 and IP-10/MIG in *Helicobacter pylori* gastritis. Clin. Exp. Immunol. 122 (2), 192–199. 10.1046/j.1365-2249.2000.01374.x 11091274 PMC1905774

[B13] Emdin C. A. Khera A. V. Kathiresan S. (2017). Mendelian randomization. JAMA-J. Am. Med. Assoc. 318 (19), 1925–1926. 10.1001/jama.2017.17219 29164242

[B14] Gibson J. A. Hahn H. P. Shahsafaei A. Odze R. D. (2011). MUC expression in hyperplastic and serrated colonic polyps: lack of specificity of MUC6. Am. J. Surg. Pathol. 35 (5), 742–749. 10.1097/PAS.0b013e31821537a2 21490447

[B15] Godos J. Biondi A. Galvano F. Basile F. Sciacca S. Giovannucci E. L. (2017). Markers of systemic inflammation and colorectal adenoma risk: meta-analysis of observational studies. World J. Gastroenterol. 23 (10), 1909–1919. 10.3748/wjg.v23.i10.1909 28348498 PMC5352933

[B16] Gong Y. Huang L. Cheng W. Li X. Lu J. Lin L. (2014). Roles of interleukin-9 in the growth and cholecystokinin-induced intracellular calcium signaling of cultured interstitial cells of Cajal. PLoS One 9 (4), e95898. 10.1371/journal.pone.0095898 24755995 PMC3995924

[B17] Goswami R. Kaplan M. H. (2011). A brief history of IL-9. J. Immunol. 186 (6), 3283–3288. 10.4049/jimmunol.1003049 21368237 PMC3074408

[B18] Gounaris E. Heiferman M. J. Heiferman J. R. Shrivastav M. Vitello D. Blatner N. R. (2015). Zileuton, 5-lipoxygenase inhibitor, acts as a chemopreventive agent in intestinal polyposis, by modulating polyp and systemic inflammation. PLoS One 10 (3), e0121402. 10.1371/journal.pone.0121402 25747113 PMC4351892

[B19] Grivennikov S. I. Greten F. R. Karin M. (2010). Immunity, inflammation, and cancer. Cell 140 (6), 883–899. 10.1016/j.cell.2010.01.025 20303878 PMC2866629

[B20] Hayakawa Y. Sakitani K. Konishi M. Asfaha S. Niikura R. Tomita H. (2017). Nerve growth factor promotes gastric tumorigenesis through aberrant cholinergic signaling. Cancer Cell 31 (1), 21–34. 10.1016/j.ccell.2016.11.005 27989802 PMC5225031

[B21] Hennink S. D. van der Meulen-De J. A. Wolterbeek R. Crobach A. S. Becx M. C. Crobach W. F. (2015). Randomized comparison of surveillance intervals in familial colorectal cancer. J. Clin. Oncol. 33 (35), 4188–4193. 10.1200/JCO.2015.62.2035 26527788

[B22] Hopkins M. H. Flanders W. D. Bostick R. M. (2012). Associations of circulating inflammatory biomarkers with risk factors for colorectal cancer in colorectal adenoma patients. Biomark. Insights. 7, 143–150. 10.4137/BMI.S10092 23170065 PMC3498968

[B23] Jass J. R. (2004). Hyperplastic polyps and colorectal cancer: is there a link? Clin. Gastroenterol. Hepatol. 2 (1), 1–8. 10.1016/s1542-3565(03)00284-2 15017625

[B24] Karnezis T. Farnsworth R. H. Harris N. C. Williams S. P. Caesar C. Byrne D. J. (2019). CCL27/CCL28-CCR10 chemokine signaling mediates migration of lymphatic endothelial cells. Cancer Res. 79 (7), 1558–1572. 10.1158/0008-5472.CAN-18-1858 30709930

[B25] Khoshakhlagh P. Bahrololoumi-Shapourabadi M. Mohammadirad A. Ashtaral-Nakhai L. Minaie B. Abdollahi M. (2007). Beneficial effect of phosphodiesterase-5 inhibitor in experimental inflammatory bowel disease; molecular evidence for involvement of oxidative stress. Toxicol. Mech. Methods. 17 (5), 281–288. 10.1080/15376510601003769 20020951

[B26] Klemke L. De Oliveira T. Witt D. Winkler N. Bohnenberger H. Bucala R. (2021). Hsp90-stabilized MIF supports tumor progression via macrophage recruitment and angiogenesis in colorectal cancer. Cell Death Dis. 12 (2), 155. 10.1038/s41419-021-03426-z 33542244 PMC7862487

[B27] Kővári B. Kim B. H. Lauwers G. Y. (2021). The pathology of gastric and duodenal polyps: current concepts. Histopathology 78 (1), 106–124. 10.1111/his.14275 33382489

[B28] Kuhn K. A. Manieri N. A. Liu T. C. Stappenbeck T. S. (2014). IL-6 stimulates intestinal epithelial proliferation and repair after injury. PLoS One 9 (12), e114195. 10.1371/journal.pone.0114195 25478789 PMC4257684

[B29] Kuhn K. A. Schulz H. M. Regner E. H. Severs E. L. Hendrickson J. D. Mehta G. (2018). Bacteroidales recruit IL-6-producing intraepithelial lymphocytes in the colon to promote barrier integrity. Mucosal Immunol. 11 (2), 357–368. 10.1038/mi.2017.55 28812548 PMC5815964

[B30] Lawlor D. A. Harbord R. M. Sterne J. A. Timpson N. Davey S. G. (2008). Mendelian randomization: using genes as instruments for making causal inferences in epidemiology. Stat. Med. 27 (8), 1133–1163. 10.1002/sim.3034 17886233

[B31] Leibowitz B. J. Zhao G. Xia W. Wang Y. Ruan H. Zhang L. (2023). mTOR inhibition suppresses Myc-driven polyposis by inducing immunogenic cell death. Oncogene 42 (24), 2007–2016. 10.1038/s41388-023-02706-6 37138032 PMC10256613

[B32] Lei-Leston A. C. Murphy A. G. Maloy K. J. (2017). Epithelial cell inflammasomes in intestinal immunity and inflammation. Front. Immunol. 8, 1168. 10.3389/fimmu.2017.01168 28979266 PMC5611393

[B33] Li W. Chen F. Gao H. Xu Z. Zhou Y. Wang S. (2023). Cytokine concentration in peripheral blood of patients with colorectal cancer. Front. Immunol. 14, 1175513. 10.3389/fimmu.2023.1175513 37063892 PMC10098211

[B34] Lin W. R. Lin D. Y. Tai D. I. Hsieh S. Y. Lin C. Y. Sheen I. S. (2008). Prevalence of and risk factors for gallbladder polyps detected by ultrasonography among healthy Chinese: analysis of 34 669 cases. J. Gastroenterol. Hepatol. 23 (6), 965–969. 10.1111/j.1440-1746.2007.05071.x 17725602

[B35] Ma X. Bi E. Huang C. Lu Y. Xue G. Guo X. (2018). Cholesterol negatively regulates IL-9-producing CD8(+) T cell differentiation and antitumor activity. J. Exp. Med. 215 (6), 1555–1569. 10.1084/jem.20171576 29743292 PMC5987919

[B36] Morey P. Pfannkuch L. Pang E. Boccellato F. Sigal M. Imai-Matsushima A. (2018). *Helicobacter pylori* depletes cholesterol in gastric glands to prevent interferon gamma signaling and escape the inflammatory response. Gastroenterology 154 (5), 1391–1404. 10.1053/j.gastro.2017.12.008 29273450

[B37] Morris K. T. Khan H. Ahmad A. Weston L. L. Nofchissey R. A. Pinchuk I. V. (2014). G-CSF and G-CSFR are highly expressed in human gastric and colon cancers and promote carcinoma cell proliferation and migration. Br. J. Cancer. 110 (5), 1211–1220. 10.1038/bjc.2013.822 24448357 PMC3950854

[B38] Nowarski R. Jackson R. Gagliani N. de Zoete M. R. Palm N. W. Bailis W. (2015). Epithelial IL-18 equilibrium controls barrier function in colitis. Cell 163 (6), 1444–1456. 10.1016/j.cell.2015.10.072 26638073 PMC4943028

[B39] Palmer T. M. Lawlor D. A. Harbord R. M. Sheehan N. A. Tobias J. H. Timpson N. J. (2012). Using multiple genetic variants as instrumental variables for modifiable risk factors. Stat. Methods Med. Res. 21 (3), 223–242. 10.1177/0962280210394459 21216802 PMC3917707

[B40] Petersen C. P. Meyer A. R. De Salvo C. Choi E. Schlegel C. Petersen A. (2018). A signalling cascade of IL-33 to IL-13 regulates metaplasia in the mouse stomach. Gut 67 (5), 805–817. 10.1136/gutjnl-2016-312779 28196875 PMC5681443

[B41] Pierce B. L. Ahsan H. Vanderweele T. J. (2011). Power and instrument strength requirements for Mendelian randomization studies using multiple genetic variants. Int. J. Epidemiol. 40 (3), 740–752. 10.1093/ije/dyq151 20813862 PMC3147064

[B42] Poffenberger M. C. Metcalfe-Roach A. Aguilar E. Chen J. Hsu B. E. Wong A. H. (2018). LKB1 deficiency in T cells promotes the development of gastrointestinal polyposis. Science 361 (6400), 406–411. 10.1126/science.aan3975 30049881

[B43] Ren F. Jin Q. Liu T. Ren X. Zhan Y. (2023). Causal effects between gut microbiota and IgA nephropathy: a bidirectional Mendelian randomization study. Front. Cell. Infect. Microbiol. 13, 1171517. 10.3389/fcimb.2023.1171517 37201114 PMC10185820

[B44] Rokavec M. öner M. G. Hermeking H. (2016). lnflammation-induced epigenetic switches in cancer. Cell. Mol. Life Sci. 73 (1), 23–39. 10.1007/s00018-015-2045-5 26394635 PMC11108555

[B45] Sakaue S. Kanai M. Tanigawa Y. Karjalainen J. Kurki M. Koshiba S. (2021). A cross-population atlas of genetic associations for 220 human phenotypes. Nat. Genet. 53 (10), 1415–1424. 10.1038/s41588-021-00931-x 34594039 PMC12208603

[B46] Sasaki Y. Takeda H. Sato T. Orii T. Nishise S. Nagino K. (2012). Serum Interleukin-6, insulin, and HOMA-IR in male individuals with colorectal adenoma. Clin. Cancer Res. 18 (2), 392–399. 10.1158/1078-0432.CCR-11-0896 22048241

[B47] Song M. Sasazuki S. Camargo M. C. Shimazu T. Charvat H. Yamaji T. (2018). Circulating inflammatory markers and colorectal cancer risk: a prospective case-cohort study in Japan. Int. J. Cancer. 143 (11), 2767–2776. 10.1002/ijc.31821 30132835 PMC6235711

[B48] Sonnenberg A. Genta R. M. (2015). Prevalence of benign gastric polyps in a large pathology database. Dig. Liver Dis. 47 (2), 164–169. 10.1016/j.dld.2014.10.004 25458775

[B49] Suzuki T. Yoshinaga N. Tanabe S. (2011). Interleukin-6 (IL-6) regulates claudin-2 expression and tight junction permeability in intestinal epithelium. J. Biol. Chem. 286 (36), 31263–31271. 10.1074/jbc.M111.238147 21771795 PMC3173073

[B50] Tete S. Saggini A. Maccauro G. Rosati M. Conti F. Cianchetti E. (2012). Interleukin-9 and mast cells. J. Biol. Regul. Homeost. Agents. 26 (3), 319–326. 23034251

[B51] Thanan R. Oikawa S. Yongvanit P. Hiraku Y. Ma N. Pinlaor S. (2012). Inflammation-induced protein carbonylation contributes to poor prognosis for cholangiocarcinoma. Free Radic. Biol. Med. 52 (8), 1465–1472. 10.1016/j.freeradbiomed.2012.01.018 22377619

[B52] Tran L. S. Ying L. D'Costa K. Wray-Mccann G. Kerr G. Le L. (2023). NOD1 mediates interleukin-18 processing in epithelial cells responding to *Helicobacter pylori* infection in mice. Nat. Commun. 14 (1), 3804. 10.1038/s41467-023-39487-1 37365163 PMC10293252

[B53] Vila M. Lladó L. Ramos E. (2018). Management and treatment of gallbladder polyps. Med. Clin. 150 (12), 487–491. 10.1016/j.medcli.2017.12.003 29426789

[B54] Wang S. Yang Z. Sha F. Qi X. He Z. Szeto C. H. (2023). Prevalence of incidental colorectal cancer and polyps in autopsies of different populations: a systematic review with meta-regression analysis. Eur. J. Epidemiol. 38 (9), 939–955. 10.1007/s10654-023-01041-0 37634229

[B55] Wang X. Wang X. Gong Y. Chen X. Zhong D. Zhu J. (2022). Appraising the causal association between systemic iron status and heart failure risk: a mendelian randomisation study. Nutrients 14 (16), 3258. 10.3390/nu14163258 36014764 PMC9412602

[B56] Wang Z. F. Ma D. G. Zhu Z. Mu Y. P. Yang Y. Y. Feng L. (2017). Astragaloside IV inhibits pathological functions of gastric cancer-associated fibroblasts. World J. Gastroenterol. 23 (48), 8512–8525. 10.3748/wjg.v23.i48.8512 29358859 PMC5752711

[B57] Wennmacker S. Z. Lamberts M. P. Di Martino M. Drenth J. P. Gurusamy K. S. van Laarhoven C. J. (2018). Transabdominal ultrasound and endoscopic ultrasound for diagnosis of gallbladder polyps. Cochrane Database Syst. Rev. 8 (8), CD012233. 10.1002/14651858.CD012233.pub2 30109701 PMC6513652

[B58] Xu A. Zhang Y. Hu H. Zhao G. Cai J. Huang A. (2017). Gallbladder polypoid-lesions: what are they and how should they be treated? A single-center experience based on 1446 cholecystectomy patients. J. Gastrointest. Surg. 21 (11), 1804–1812. 10.1007/s11605-017-3476-0 28695432

[B59] Ye J. Zhu Y. Khan W. I. Van Snick J. Huizinga J. D. (2006). IL-9 enhances growth of ICC, maintains network structure and strengthens rhythmicity of contraction in culture. J. Cell. Mol. Med. 10 (3), 687–694. 10.1111/j.1582-4934.2006.tb00428.x 16989728 PMC3933150

[B60] Yu B. Xiang L. Peppelenbosch M. P. Fuhler G. M. (2023). Overlapping cytokines in *H. pylori* infection and gastric cancer: a tandem meta-analysis. Front. Immunol. 14, 1125658. 10.3389/fimmu.2023.1125658 37006300 PMC10050690

[B62] Yang T. Wang R. Zhang J. Bao C. Zhang J. Li R. (2020). Mechanism of berberine in treating Helicobacter pylori induced chronic atrophic gastritis through IRF8-IFN-γ signaling axis suppressing. Life. Sci. 248, 117456. 10.1016/j.lfs.2020.117456 32097666

[B61] Zuber V. Colijn J. M. Klaver C. Burgess S. (2020). Selecting likely causal risk factors from high-throughput experiments using multivariable Mendelian randomization. Nat. Commun. 11 (1), 29. 10.1038/s41467-019-13870-3 31911605 PMC6946691

