# Supplementary figures and images for: Causal links between circulatory inflammatory cytokines and risk of digestive polyps: a Mendelian randomization analysis
Source: Front Pharmacol. 2024 Oct 8;15:1405503. doi: 10.3389/fphar.2024.1405503 (PMC11493649; doi:10.3389/fphar.2024.1405503)

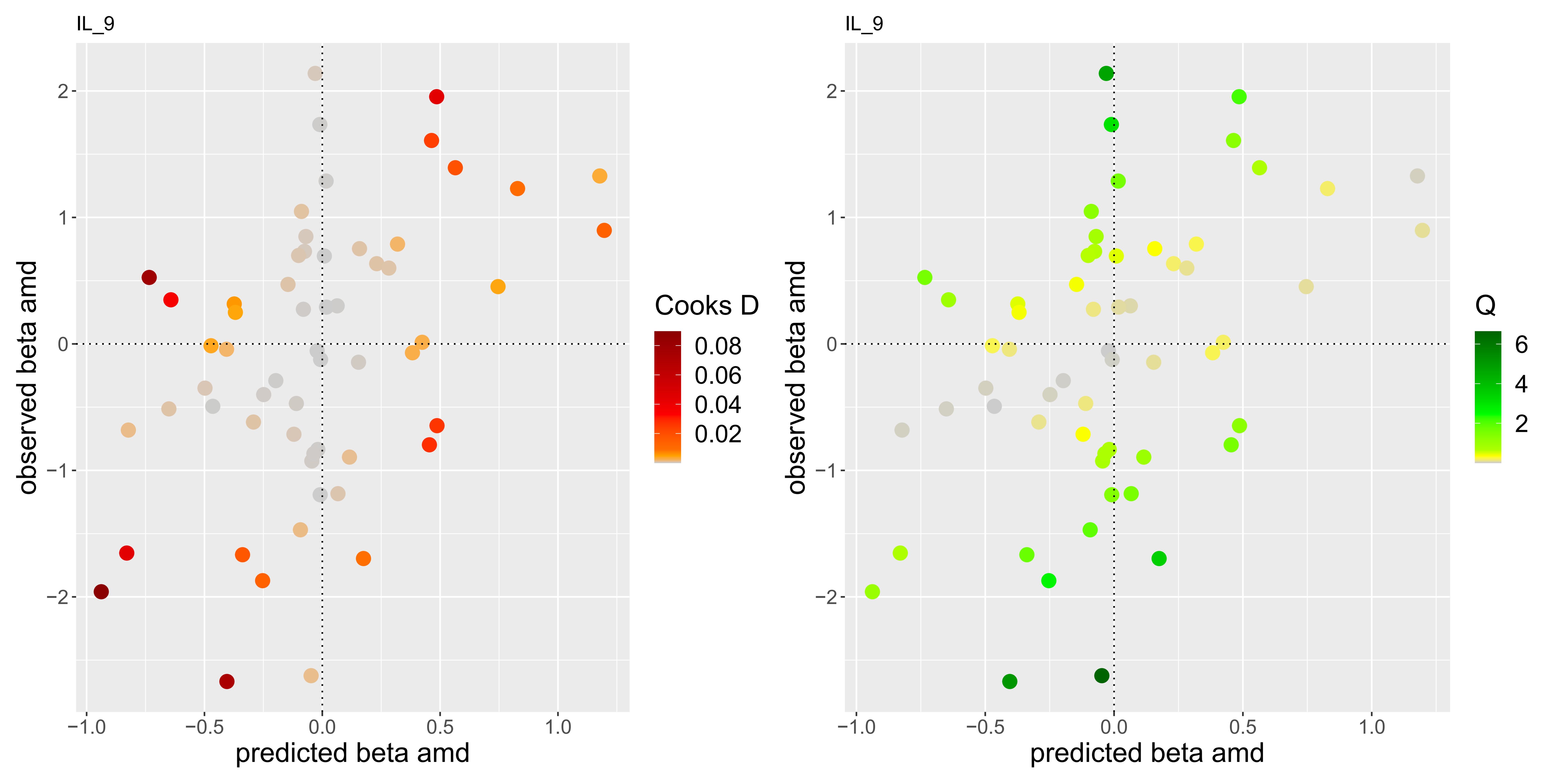

Supplement: Supplementary file 1 [file Image3.JPEG]

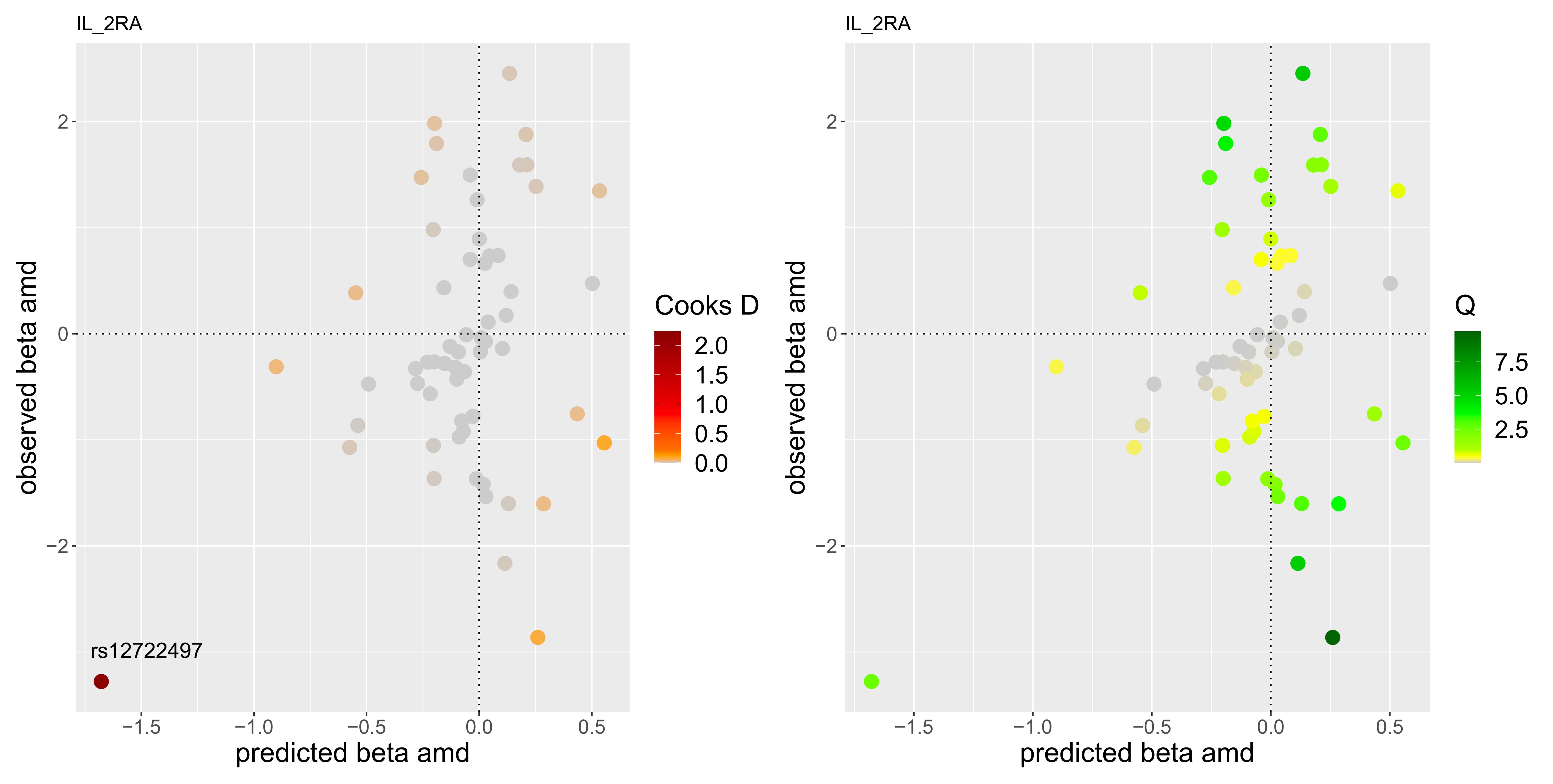

Supplement: Supplementary file 2 [file Image1.JPEG]

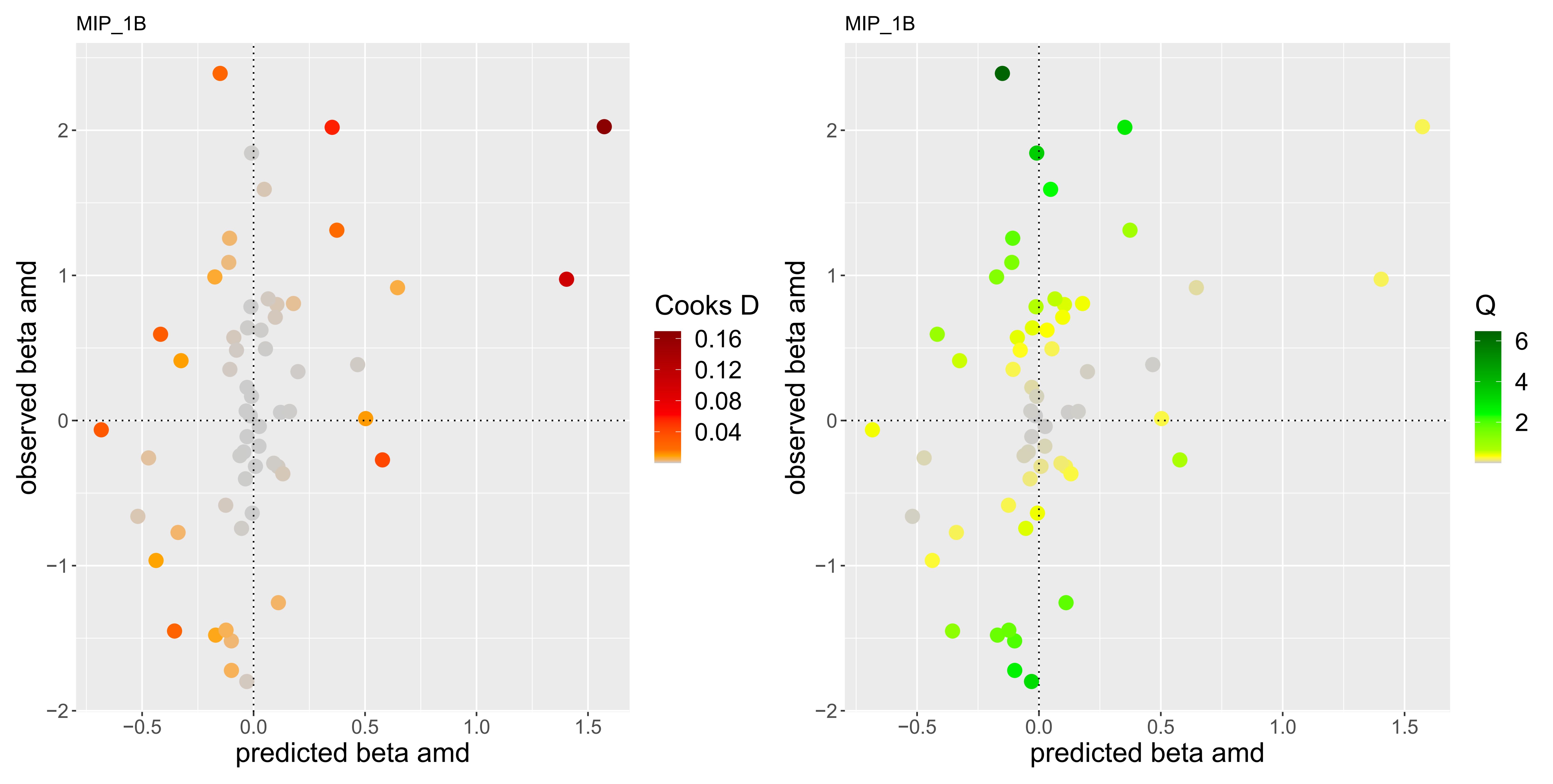

Supplement: Supplementary file 3 [file Image2.JPEG]
